# Analysis of factors associated with IUI pregnancy outcomes in elderly and young patients

**DOI:** 10.1186/s12905-024-02934-2

**Published:** 2024-02-03

**Authors:** Chunmei- Yu, Lijing- Bai, Jian mei-Zhou, Xiao yu-Wang, Li Chen, Jinghua- Zhang

**Affiliations:** Department of Reproduction, Changzhou Maternity and Child Health Care Hospital, Changzhou Medical Center, Nanjing Medical University, Changzhou, Jiangsu 213000 China

**Keywords:** Intrauterine insemination (IUI), Pregnancy outcomes, Cycle number, Young women, Aged women

## Abstract

**Objective:**

The objective of this study was to investigate the correlation between various factors and the clinical outcomes of Intrauterine Insemination (IUI) in both young and aged patients, aiming to provide a theoretical basis for clinical consultations.

**Methods:**

This retrospective analysis examined a total of 4,221 IUI cycles conducted at the Reproductive Center of Changzhou Maternal and Child Health Hospital between January 2016 and December 2020. The patients were categorized into two groups based on age: the elder group (≥ 35 years) and the young group (< 35 years).

**Results:**

The findings of this study revealed a significant association between woman’s age and BMI with pregnancy outcomes (0.93, 95% CI: 0.89–0.97) (1.04, 95% CI: 1.01–1.06). Moreover, in young women, both age and Body Mass Index (BMI)were found to be related to pregnancy outcomes (0.97, 95% CI: 0.89–0.97) (1.08, 95% CI: 1.01–1.06). Additionally, BMI and the number of cycles in aged IUI patients were found to be associated with pregnancy outcomes. The pregnancy rate in the second cycle was approximately 1.9 times higher than that in the first cycle (1.9, 95% CI: 0.97–3.77), and in the third cycle, it was approximately 3 times higher than that in the first cycle (3.04, 95% CI: 1.43–6.42).

**Conclusions:**

In conclusion, there is an association between woman’s age and BMI and the clinical outcomes of IUI. However, the number of cycles did not affect the pregnancy outcomes in young women. Conversely, in elder women, the number of cycles was found to be related to the IUI pregnancy outcomes, with significantly higher pregnancy rates observed in the second and third cycles compared to the first cycle.

## Introduction

Approximately 10% of couples worldwide experience infertility [1]. While assisted reproductive technology (ART) is a common and effective treatment for infertile couples, intrauterine insemination (IUI) remains a popular option due to its effectiveness, affordability, and safety [2]. IUI is primarily used to address mild male factor infertility, anovulation, endometriosis, and unexplained infertility. Among these, patients with anovulation tend to have the best treatment outcomes [3]. Despite advancements in IUI techniques, such as ovulation stimulation, the clinical pregnancy rate of IUI still lags behind that of in vitro fertilization (IVF). Numerous studies have identified several factors associated with the pregnancy outcomes of IUI, including female age, female BMI, duration of infertility, and dominant follicles [4, 5].

Many scholars and experts in the field of assisted reproductive technology recommend considering IVF treatment after three to four cycles of IUI. This recommendation is based on the observation that the majority of pregnancies occur within the first four cycles, and subsequent cycles may offer little additional benefit [6–8]. Currently, there is a consensus among experts in assisted reproductive technology that IUI should be attempted for three to four cycles, and if unsuccessful, patients should consider transitioning to IVF treatment. This approach is widely adopted as a diagnostic and treatment guideline in most reproductive centers. However, it is important to note that the optimal number of IUI cycles may vary for each individual patient. Due to the variability in patient characteristics and underlying infertility issues, a fixed recommendation of three or four cycles may not be appropriate for everyone. Clinical research has not yet provided guidelines that can be easily adapted to address the specific needs and challenges of each patient. Therefore, it is crucial for healthcare providers to carefully evaluate each patient’s unique circumstances and consider personalized treatment plans based on factors such as age, fertility history, and response to previous cycles.

Age is indeed a significant indicator of female fertility and plays a crucial role in assisted reproductive technology [9]. As women age, their fertility declines due to factors such as diminished ovarian reserve and an increased risk of chromosomal abnormalities in oocytes (aneuploidy) [10, 11]. It is uncertain whether factors related to pregnancy outcomes differ between older and younger patients undergoing IUI. Therefore, the purpose of the study you mentioned is to explore the correlation between various factors and IUI pregnancy outcomes in both young and older patients. By examining these associations, the study aims to provide a theoretical basis for clinical consultations and guidance when considering IUI as a treatment option.

## Materials and methods

### Study design and participant

This study is a retrospective analysis of IUI cycles (4221)at the reproductive center of the Changzhou Maternal and Child Health Hospital from January 2016 to December 2020.

The patients were categorized into two groups according to age, the elder group (≥ 35 years)and the young group (<35 years).

The exclusion criteria were: (1) chromosomal abnormalities in either partner of the couple, (2) presence of ovarian cysts at the start of the ovarian stimulation procedure, (3) abnormal uterine cavity, ovarian deficiency, or other organic diseases;(4) The patients with PCOS ,BMI ≥ 30 kg/m^2^ .

The study and the protocols used were approved by the Ethics Committee of the Changzhou Maternal and Child Health Care Hospital (17,020,490,718).Written informed consent was obtained from the infertility couple.

### Ovarian stimulation and IUI protocol

According to the literature reports, the pregnancy rate of IUI cycle is higher than that of natural cycle, and most patients in our center were performed the Letrozole (LE, Jiangsu Hengrui Pharmaceutical Company), human menopausal gonadotropin (hMG,Livzon Pharm), or hMG with LE.The dosages were determined by BMI, ovarian reserve assessment, and the historical ovarian reactivity. The monitor of follicles and endometrium were performed by the transvaginal ultrasound (TVUS). IUI was canceled if more than three dominant follicles. Ovulation was triggered with hCG (hCG, Livzon Pharm) 10,000 IU or Triptorelinacetate (GnRH-a,Ferring Pharmaceuticals) 0.1 mg when the mean diameter reached 18 mm. IUI performed after 26–36 h of trigger injection. The man’s semen is removed by gradient centrifugation in the laboratory and then injected directly into the woman’s uterus through a soft IUI catheter. Luteal phase support was provided with Progesterone Soft Capsules 100 mg twice daily for 14 days after ovulation was determined.

### Sperm preparation

Sperm parameters strictly follow the fifth WHO edition(2010).Patients with 2–5 days of abstinence ejaculated semen into a sterilized cup by masturbation. After liquefaction, 10 ml of semen sample was drawn on the slide to roughly estimate sperm parameters (concentration and motile) including the percentage of progressive motility (PR) sperm, non-PR (NP) and immobile (IM) sperm. Te remaining sample was placed on a density gradient column consisting of 1.5 ml of lower layer and 1.5 ml of upper layer (Irvine, USA) and centrifuged at.

1750 rpm for 15 min. After that, the sperm pellet was re-suspended in 2.7 ml of medium and and centrifuged at 1500 rpm for 9 min after absorbing the supernatant and leaving 0.5 ml for mixing.Sperm analysis was performed by the same laboratory technician according to the a quality control program.

### Outcome assessment and follow-up visit

The blood test carried out to determine pregnancy. TVUS was performed one month after IUI. Professionals followed up the condition of pregnancy and childbirth.

### Statistical analysis

Statistical analyses were performed using SPSS software version 26.0 (IBM, Armonk, NY, USA). Quantitative variables with normal distribution were expressed as mean ± standard deviation, and Students’ t test was used to compare means. Quantitative variables with abnormal distribution or heterogeneous variance were expressed as M(P25,P75), and the median was compared by Mann-Whitney U-test. Chi-square test was used to compare the differences between the two groups. Fisher exact test was used to compare differences in rates when the expected count was < 5or the total sample size was < 40.*P* < 0.05 was considered statistically significant. Multi-factor logistic regression analysis of factors associated with IUI pregnancy outcomes was presented as forest maps,an OR value of 1 indicates no correlation, an OR value greater than 1 indicates a positive correlation, and an OR value less than 1 indicates a negative correlation.

## Results

A total of 2110 couples underwent 4221 cycles in this study. The mean age was 30 years old, and the mean duration of infertility was about three years. The clinical pregnancy rate (CPR) was 13.6% per cycle ; The abortions rate about 10.3% and the ectopic pregnancy rate about 1.84%; 90.8% children were singleton pregnancies and 4.52% children were twins.The proportion of the first cycle is about 49.6%(2093/4221),the proportion of the second cycle is 33.4%(1410/4221) and the proportion of the third cycle is 13.0%(550/4221).

### Compared the cycle characteristics of patients between the pregnancy and non-pregnancy group

The couple age in the pregnancy group is younger than the non-pregnancy group(*P*<0.001) (*P* = 0.003),and the BMI in the pregnancy group is higher than the non-pregnancy group(*P* = 0.003).The normal morphology rate in the pregnancy group was higher than the non-pregnancy group (*P* = 0.023) .There was no difference in other indicators, including the woman’s infertility diagnosis, infertility years, infertility types, treatment options, and male body mass index and other semen parameters (Table 1).

**Table 1:** Compared the cycle characteristics of patients between the pregnancy and non-pregnancy group

Variables	Non-pregnant(*n* = 3646)	Pregnant(*n* = 575)	*P*
Female age (years)	30.86 ± 3.31	30.20 ± 3.29	< 0.001
AMH(ng/ml)	3.02 [1.84,5.26]	3.28 [1.81,5.96]	0.191
Male age (years)	32.04 ± 4.03	31.50 ± 3.92	0.003
Female BMI( kg/m2)	22.53 ± 3.51	23.00 ± 3.71	0.003
Male BMI( kg/m2)	28.61 ± 93.92	25.37 ± 4.05	0.600
Cycle number(n)			
1	1802 (49.42)	291 (50.61)	0.859
2	1223 (33.54)	187 (32.52)	
3	621 (17.03)	97 (16.87)	
Duration of infertility(years)	3.00 [2.00, 4.00]	2.00 [2.00, 4.00]	0.063
Female diagnosis (%)			
Male	1165 (31.95)	173 (30.31)	0.12
Unexplained infertility	1738 (47.69)	261(45.45)	
Endometriosis	69 (1.90)	8 (1.48)	
Ovulation disorder	600 (16.46)	125 (21.77)	
Infertility type (%)			
Primary infertility	2750 (75.43)	434 (75.35)	1.000
Second infertility	896 (24.57)	142 (24.65)	
Number of the dominant follicle (n)	1(1,2)	1(1,2)	0.66
Endometial thickness(cm)	0.87 ± 0.15	0.94 ± 0.18	0.310
Treatment methods (%)			
Ovarian stimulation cycle	3504 (96.11)	556 (96.70)	0.569
Natural cycle	142 (3.89)	19 (3.30)	
Total sperm count(million)	159.76 [98.49, 255.20]	154.90 [96.50, 253.98]	0.707
Normal morphology rate(%)	4.40 [4.10, 4.80]	4.40 [4.10, 4.90]	0.023
Progressive motility(million)	42.12 ± 16.86	42.91 ± 17.13	0.293
Total motile sperm count(million)	61.54 [31.95, 111.31]	61.50 [32.31, 115.36]	0.805

### Compared the cycle characteristics of young patients between the pregnancy and non-pregnancy group

The couple age in the pregnancy group is younger than the non-pregnancy group(*P* < 0.001)(*P* = 0.04),and the women BMI in the pregnancy group is higher than the non-pregnancy group(*P* = 0.01);The duration of infertility in the pregnancy group is lower than the in the pregnancy group (*P* = 0.033).The normal morphology rate in the pregnancy group was higher than the non-pregnancy group (*P* = 0.01).The proportion of the secondary infertility in the pregnancy group higher than the non-pregnancy group.There was no difference in other indicators, including the woman’s infertility diagnosis, treatment options, and male body mass index and other semen parameters (Table 2).

**Table 2:** Compared the cycle characteristics of young patients between the pregnancy and non-pregnancy group

Variables	Non-pregnant(*n* = 3177)	Pregnant(*n* = 522)	*P*
Female age (years)	29.99 ± 2.49	29.54 ± 2.62	< 0.001
AMH(ng/ml)	3.98(1.85,5.34)	4.11(1.95,5.82)	0.724
Male age (years)	31.17 ± 3.13	30.87 ± 3.26	0.040
Female BMI( kg/m2)	22.51 ± 3.55	22.95 ± 3.72	0.010
Male BMI( kg/m2)	29.11 ± 100.50	25.45 ± 4.12	0.599
Cycle number (n)			
1	1562 (49.17)	274 (52.49)	0.355
2	1065 (33.52)	166 (31.80)	
3	550 (17.31)	82 (15.71)	
Duration of infertility (years)	3.00 [2.00, 4.00]	2.00 [2.00, 3.00]	0.033
Female diagnosis (%)			
Male	1026 (32.32)	157 (30.16)	0.13
Unexplained infertility	1450 (45.65)	251 (48.16)	
Endometriosis	62 (1.95)	6 (1.22)	
Ovulation disorder	543 (17.08)	102 (19.45)	
Infertility type (%)			
Primary infertility	2482 (78.12)	397 (75.91)	0.283
Second infertility	695 (21.88)	126 (24.09)	
Number of the dominant follicle (n)	1(1,2)	1(1,2)	0.75
Endometial thickness(cm)	0.84 ± 0.16	0.95 ± 0.17	0.33
Treatment methods (%)	3053 (96.10)	503 (96.36)	0.868
Ovarian stimulation cycle			
Natural cycle	124 (3.90)	19 (3.64)	
Total sperm count(million)	159.67 [99.00, 253.12]	158.24 [97.98, 254.94]	0.990
Normal morphology rate(%)	4.40 [4.10, 4.80]	4.40 [4.10, 4.90]	0.010
Progressive motility(million)	42.35 ± 16.93	42.98 ± 16.97	0.424
Total motile sperm count(million)	62.25 [31.97, 112.34]	62.83 [33.11, 117.44]	0.629

### Compared the cycle characteristics of aged patients between the pregnancy and non-pregnancy group

The women BMI in the pregnancy group is higher than the non-pregnancy group(*P* = 0.03).There was significant difference in the ratio of cycles between the two groups(*P* = 0.011).There was no difference in other indicators, including the woman’s infertility diagnosis, infertility years, treatment options, and male body mass index and semen parameters (Table 3).

**Table 3:** Compared the cycle characteristics of aged patients between the pregnancy and non-pregnancy group

Variables	Non-pregnant(*n* = 469)	Pregnant(*n* = 53)	P
Female age (years)	36.78 ± 1.77	36.70 ± 1.85	0.756
AMH(ng/ml)	3.01 [1.54,5.26]	3.19 [1.77,5.82]	0.38
Male age (years)	37.92 ± 4.51	37.74 ± 4.45	0.776
Female BMI( kg/m2)	22.64 ± 3.23	23.53 ± 3.57	0.030
Male BMI( kg/m2)	25.23 ± 3.45	24.63 ± 3.29	0.437
Cycle number (%)			
1	240 (51.17)	17 (32.08)	0.011
2	158 (33.69)	21 (39.62)	
3	71 (15.14)	15 (28.30)	
Duration of infertility (years)	3.00 [2.00, 5.00]	3.00 [2.00, 5.00]	0.300
Female diagnosis (%)			
Unexplained infertility	152(32.45)	20 (37.23)	0.761
Male	210 (44.80)	22 (41.54)	
Endometriosis	7 (1.53)	2 (3.85)	
Ovulation disorder	52 (11.22)	8 (15.38)	
Infertility type (%)			
Primary infertility	268 (57.14)	35 (66.04)	0.273
Second infertility	201 (42.86)	18 (33.96)	
Number of the dominant follicle (n)	1(1,2)	1(1,2)	0.82
Endometial thickness(cm)	0.87 ± 0.16	0.90 ± 0.17	0.43
Treatment methods (%)			
Ovarian stimulation cycle	451 (96.16)	53 (100.00)	0.292
Natural cycle	18 (3.84)	0 (0.00)	
Total sperm count(million)	162.90 [92.10, 258.45]	129.90 [77.76, 248.00]	0.215
Normal morphology rate(%)	4.30 [4.10, 4.80]	4.20 [3.70, 4.90]	0.414
Progressive motility(million)	40.57 ± 16.36	42.22 ± 18.76	0.493
Total motile sperm count(million)	57.91 [31.84, 99.84]	50.64 [27.41, 83.84]	0.364

### Multivariate logistic regression analysis of factors associated with pregnancy outcomes in younger and aged IUI patients

**Fig. 1:**
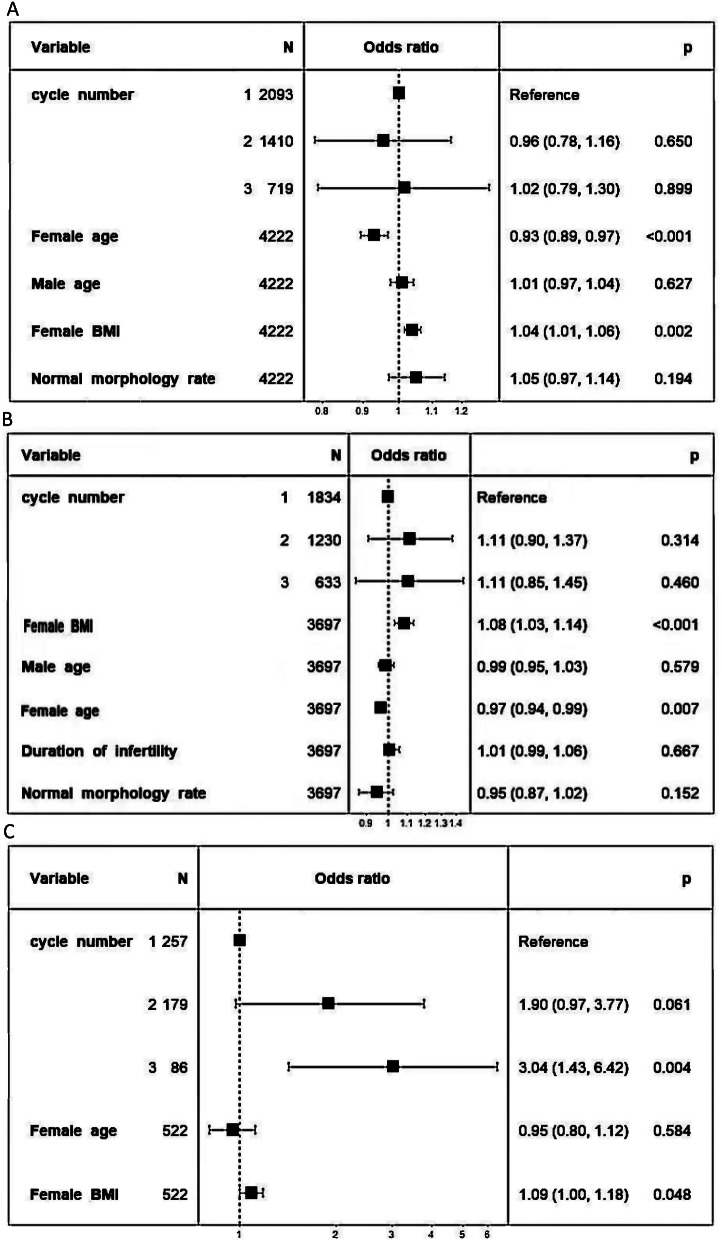
Multivariate logistic regression analysis of factors associated with pregnancy outcomes in total IUI patients ,younger and aged IUI patients (total patients (**A**) younger patients (**B**) aged patients (**C**))

Multivariate logistic regression analysis showed that the age and BMI of total IUI patients are closed related to the pregnancy outcome(*P*<0.001)(*P* = 0.002),the number of cycles were not associated with the pregnancy outcome(Fig. 1-A); In the younger group reached the same conclusion, with women age having a negative effect on IUI pregnancy outcomes and women BMI having a positive effect(*P*<0.001)(Fig. 1-B).The BMI and the cycle number of aged IUI patients are related with the pregnancy outcome, the pregnancy rate of the second cycle is about 1.9 times that of the first cycle(*P* = 0.06), and the third cycle is about 3 times that of the first cycle (*P* = 0.06)(Fig. 1-C).

## Discussions

The present study revealed significant associations between women’s age and BMI with pregnancy outcomes. Additionally, the BMI of women and the time of IUI cycles were found to be associated with the pregnancy outcome among older women. These results highlight the importance of considering both age and BMI as potential factors influencing pregnancy outcomes in different age groups of women.

Our study supports previous research findings that the age of women is closely related to the pregnancy outcome in the general population. It is well-known that female fertility gradually declines starting from the age of 30, with a significant drop in pregnancy rates after the age of 40. Consequently, it is generally not recommended to undergo IUI after the age of 40 [12, 13]. A randomized trial comparing the clinical pregnancy rates of natural conception and ovulation induction with IUI cycles in couples with unexplained or male factor infertility found age to be the sole predictor. The study reported a 50% lower chance of pregnancy for 38-year-old women compared to those who were 28 years old [10]. In women, as they age, telomerase shortening and age-related mitochondrial mtDNA instability can lead to the accumulation of mtDNA mutations in oocytes, resulting in a decline in the quality of the ovarian reserve and a decrease in the implantation rate [14, 15]. Our study also confirmed a negative correlation between age and the pregnancy outcome of IUI, with the effect being more pronounced in the younger group and less pronounced in the older group. This may be attributed to the fact that patients in the older group are typically over 35 years old, where the age range is relatively concentrated, and ovarian function experiences a sharp decline. Therefore, the slight difference of a year or two in age may not have a significant impact due to the limited variation among patients in this age group.

The findings of our study indicate that women with higher BMI had higher pregnancy rates compared to women with lower BMI, regardless of their age group. This consistent relationship between BMI and the pregnancy outcome of IUI is noteworthy.It is known that cholesterol in fat cells serves as the raw material for the synthesis of sex hormones. Women with low BMI and low subcutaneous fat have lower cholesterol content and lower levels of sex hormones in their bodies. This can have a dual impact on ovarian function and endometrial development, ultimately affecting the pregnancy outcome of IUI [16]. Research has shown that women with relatively low BMI have lower concentrations of serum hCG and progesterone, which can affect endometrial thickness and lead to early pregnancy loss [17]. Studies have also found that being underweight is associated with infertility due to anovulation and hypothalamic amenorrhea. Underweight patients have an increased risk of delivering small-for-gestational-age babies, especially when undergoing ovulation induction [18, 19]. On the other hand, there is a positive correlation between BMI and endometrial thickness. However, obesity increases reproductive risks, including infertility due to menstrual dysfunction and oligo-anovulation [20–23]. In our study, the average BMI of patients was around 23, with most patients falling within the normal BMI range, and only a relatively small proportion of patients being obese. Therefore, within the normal BMI range, higher BMI values were found to be more favorable for the outcome of IUI in our study.

Most experts recommend considering a switch to in vitro fertilization (IVF) after three or four intrauterine insemination (IUI) cycles because the majority of pregnancies resulting from IUI occur within the first few cycles. Studies have demonstrated that up to 95% of pregnancies achieved through IUI with gonadotropins or clomiphene citrate (CC) occur within the first three cycles, and up to 98% occur within the first four cycles [8]. However, these recommendations may not meet the individual needs of patients, especially considering factors such as age. It is important for doctors and patients to understand the potential benefits of continuing with different numbers of IUI cycles, and there is no theoretical support for a specific cutoff. Our study found that the number of IUI cycles was not related to pregnancy outcomes in the younger group, but it was closely related to outcomes in the older group. Previous research has also confirmed that women under 30 years old required an average of 1.99 cycles to conceive, while those over 40 years old required an average of 2.24 cycles [3]. Comparing the second cycle to the first cycle, the pregnancy rate was 1.9 times higher, and the third cycle had a pregnancy rate over 3 times higher than the first cycle. In other words, for younger patients, increasing the number of IUI cycles may not significantly improve the pregnancy outcome. However, for older patients, it may be beneficial to increase the number of IUI cycles to improve the pregnancy rate. It is possible that for younger patients, switching to IVF after one or two IUI failures may be sufficient, while for older patients, increasing the number of IUI cycles to four before considering IVF could be considered. However, it is important to note that older patients also face the risk of premature ovarian failure, so a comprehensive assessment is needed before making a decision. This assessment should consider factors such as the patient’s age, ovulation status, cost of treatment, and the patient’s goals.

When interpreting the results of the study, it is important to consider both the advantages and limitations. Some of the advantages of the study include analyzing the correlation between the number of IUI cycles and outcomes in patients of different ages, which has not been extensively studied before. Additionally, the study benefits from having complete medical records and accurate follow-up data. The large sample size (around 4,000) and the study period of about three years also contribute to the study’s strength.However, there are several limitations that should be acknowledged. Firstly, the study is a retrospective study conducted at a single center. While the sample size is large, and efforts were made to control for confounding factors using multivariate logistic regression, there may still be residual confounding factors. Therefore, further validation using a large multi-center sample is necessary.Secondly, due to expert consensus and strict management of assisted reproductive technology, there is a lack of data on the fourth and fifth cycles of IUI. However, the data from the first three cycles can still provide a certain theoretical basis.

In conclusion, the study found associations between women’s age and BMI with the clinical outcomes of IUI. The number of cycles in young women was not significantly associated with the pregnancy outcome in IUI. However, in older women, the number of cycles was related to the pregnancy outcome, with higher pregnancy rates observed in the second and third cycles compared to the first cycle.

## Data Availability

The data used during the current study are available from the corresponding author on reasonable request.

## Abbreviations

IUI: Intrauterine insemination
BMI: Body Mass Index
IVF: In vitro fertilization
ART: Assistant reproductive technology
HMG: Human menopausal gonadotropin
LE: Letrozole
TVUS: Transvaginal ultrasound
PR: Progressive motility sperm
CPR: Clinical pregnancy rate
